# Study on high temperature solidification behavior and crack sensitivity of Fe-Mn-C-Al TWIP steel

**DOI:** 10.1038/s41598-019-52381-5

**Published:** 2019-11-04

**Authors:** Changling Zhuang, Jianhua Liu, Changrong Li, Daowen Tang

**Affiliations:** 1 College of Materials and Metallurgy, Guizhou University, Guiyang, 550025 Guizhou China; 2 Engineering Research Institute, University of Science and Technology Beijing, Beijing, 100083 China

**Keywords:** Metals and alloys, Theory and computation

## Abstract

Fe-Mn-C-Al alloy is a new steel grade of TWIP steel developed in recent years. It has an excellent combination of elongation and tensile strength, as well as good anti-delayed fracture property. However, the crack sensitivity of this new TWIP steel has not been reported yet. In this study, differential thermal analysis (DTA) method was used, combined with professional thermodynamic software ThermoCalc to analyze the solidification behavior for Fe-Mn-C-Al alloys with different chemical compositions. Based on this, the crack sensitivity of TWIP steel is further determined. Through this study, it was found that Fe-Mn-C-Al TWIP steel may have a solidification sequence with high crack sensitivity, belonging to hypo-peritectic steel. Moreover, it was found that the carbon content has a large influence on the solidification behavior, and the manganese content also affects the solidification sequence. It can make the phase transition sequence of the solidification process change significantly, which may avoid the solidification behavior of hypo-peritectic reaction. The analysis results by thermodynamic software ThermoCalc are in good agreement with the experimental results. It displays thermoCalc can be a cost-effective way to develop Fe-Mn-C-Al TWIP steel. It is of great significance for shortening the development period of new Fe-Mn-C-Al steel grades.

## Introduction

Twinning-induced plasticity (TWIP) steel has shown both high strength and high plasticity. It is regarded as an ideal automotive structural material and exhibits a broad application prospect^1–4^. In recent years, the research on the composition and properties of TWIP steel has entered a new stage. From the initial low-carbon Fe-Mn-Si-Al TWIP steel, it developed to Fe-Mn-C TWIP steel, and then to the latest Fe-Mn-C-Al TWIP steel. The development of TWIP steel with an excellent combination of elongation and tensile strength has become a hot topic^5–10^. At present, research mainly focuses on the structure and properties of TWIP steel^11–13^. But the study on the solidification process and crack sensitivity is rare, especially for the latest Fe-Mn-C-Al TWIP steel; its solidification mode under high-temperature process has not been reported^14,15^. Due to the composition design of the new TWIP steel, this alloy may be in the range of hypo-peritectic reaction, which may undergo a peritectic transformation and prone to cause cracks and defects; this type of steel with high crack susceptibility can be classified into hypo-peritectic steel.

The hypo-peritectic steel with specific transformation sequence during solidification is responsible for pits, cracks and other defects, resulting in more defect appearances in production^16–18^. In the Fe-C equilibrium phase diagram, hypo-peritectic steel has a carbon content of 0.09 to 0.17%. Since the actual steel contains many elements, the peritectic point will change, and the determination of the hypo-peritectic steel becomes difficult^19^.

At present, carbon equivalent method is a widely used method for determining hypo-peritectic steel. It simply adds the influence value of each constituent element and converts the element content into carbon equivalent Cp^20,21^. If the value of Cp is between 0.09 and 0.17%, the steel is considered to be hypo-peritectic steel. The limitation of this approach is lack consideration of the interaction between component elements. It has a small scope of application, and may not be suitable for high alloy steel. Many researchers, like Xia *et al*.^21^, Kagawa^22^, Yamada^23^, and Blazek^24^, predicted the range of hypo-peritectic by calculation of the concentration and temperature of the critical point. Xia *et al*. gave the equivalent carbon content for peritectic point in Eq. (1)^21^.

1 $$\begin{array}{rcl}{\rm{Cp}} & = & [ \% {\rm{C}}]+0.02[ \% {\rm{Mn}}]-0.037[ \% {\rm{Si}}]\\  &  & +\,0.023[ \% {\rm{Ni}}]-0.0189[ \% {\rm{Mo}}]\\  &  & -\,0.7[ \% {\rm{S}}]+0.0414[ \% {\rm{P}}]+0.003[ \% {\rm{Cu}}]\\  &  & -\,0.0254[ \% {\rm{Cr}}]-0.0276[ \% {\rm{Ti}}]+0.7[ \% {\rm{N}}]\end{array}$$

This approach considers the influence of constituent elements on the critical point. But its limitations on steel grades are obvious and can only be applied in a limited concentration range.

Professional thermodynamic software, such as ThermoCalc or Factsage, can also be used to predict the solidification process of steel, which is based on a powerful thermodynamic database^25^. This approach can obtain satisfactory results for common steel grade, but for a new steel grade, the use of this method might result in unrealistic value. In contrast, the experimental method can accurately determine the high temperature phase transformations of the target steel. Presoly^26^ has used differential scanning calorimeter (DSC) combined with high-temperature laser scanning confocal microscope to successfully analyze the hypo-peritectic steel and obtained good results. Presoly pointed out that one DSC measurement was enough to confirm the phase transformation characteristics and determine whether it belonged to hypo-peritectic steel. But this method has higher requirements for a laboratory technician.

In this study, differential thermal analysis (DTA) experiments combined with the thermodynamic software ThermoCalc were performed to analyze TWIP steel with different chemical compositions, and to determine the solidification behavior at high temperatures. Finally, it is analyzed whether the TWIP steel with different compositions belongs to the critical range with high crack sensitivity. The results show that the solidification behavior of Fe-Mn-C-Al TWIP steel may be hypo-peritectic steel. This solidification mode can easily lead to cracks or other defects. The analysis results of the thermodynamic software ThermoCalc are in good agreement with the experimental results. The thermodynamic software ThermoCalc can be used to make a better prediction in the development of this type of steel, which is of great significance for shortening the development cycle of steel grade.

## Experimental Materials and Methods

### Experimental materials

Three different Fe-Mn-C-Al TWIP steels were prepared by vacuum smelting furnace. The chemical composition as shown in Table 1. The samples with the dimension of 50 mm*30 mm*20 mm were obtained from smelting ingot. Then the samples were further cut into 100 mg weight standard DTA samples, which were prepared for experiments after polishing.

**Table 1:** Composition of TWIP steels in the experiments, wt%.

Sample	C	Mn	Al	Si	P	Fe
TWIP A	0.13	26.3	3.42	0.03	0.004	Bal.
TWIP B	0.49	26.8	3.78	0.03	0.005	Bal.
TWIP C	0.34	21	3.34	0.03	0.005	Bal.

### Thermal analysis methods

The differential thermal analysis (DTA) method measures all characteristic temperature during thermal reaction associated with an endothermic or exothermic effect. It is a good approach to analyze physical and chemical reactions such as phase transformation, decomposition, combination and solidification^27^. In this study, DTA was used to study the phase transition and solidification modes of three TWIP steels at high temperature. A 3.0 mm × 2.2 mm × 2 mm sample was used for the experiment and the whole experimental process was performed under argon protection with a purity of 99.9999%, wherein the argon flow rate was 70 cm ^−3^ min ^−1^.

At the beginning of the experiment, it is heated to the predetermined maximum temperature at a speed of 20 to 30 K/min. The maximum temperature is generally about 40 to 60 K higher than the liquidus temperature. After that let it cool down, and then reheat it again to the maximum temperature with a certain heating rate, which can be 5 K/min, 7.5 K/min, and 10 K/min. The phase transformation temperature of the TWIP steel is determined during the heating process.

In addition, for the sake of the accuracy, experiments were carried out in the same experimental environment, including the placement position, the number of evacuation times, the degassing film, the heating schedule, etc.; the DTA instrument was calibrated with high purity cobalt. The melting point of cobalt measured twice by the DTA experiments is 1767.95 K, which is almost the same as the reported melting point of 1768.15 K (1495.0 °C)^28^. Therefore, the accuracy of experimental measurement for phase transition temperature can be guaranteed.

### CALPHAD Method - ThermoCalc

The commercial software ThermoCalc is widely used in the calculation of phase diagrams (CALPHAD), and this CALPHAD approach is based on previously measured alloy data. It can calculate thermodynamic properties or phase equilibria, and also can draw phase diagrams by adopting relevant calculation modules^29–31^. The TCFE6 Database includes many critical assessments on thermodynamic data for multi-component systems, it is applicable for various types of steels/Fe-alloys. In this research, ThermoCalc in combination with the FCFE6 database was used to calculate the solidification process of TWIP steels with different chemical composition, predict the temperature of the phase transition point, and analyze the possible solidification sequence.

## Results and Discussion

### Thermal analysis experiment results

In this study, TWIP steel samples with different chemical compositions, TWIP A, TWIP B, and TWIP C were prepared for differential thermal analysis. The enthalpy change of each sample under high temperature was recorded at a heating rate of 5 K/min, 7.5 K/min, and 10 K/min respectively, thus the temperature of the phase transition point at high temperature was obtained. Therefore, the solidification behavior of the sample during high temperature was determined. The experimental results were analyzed by NETZSCH Proteus software.

**Figure 1:**
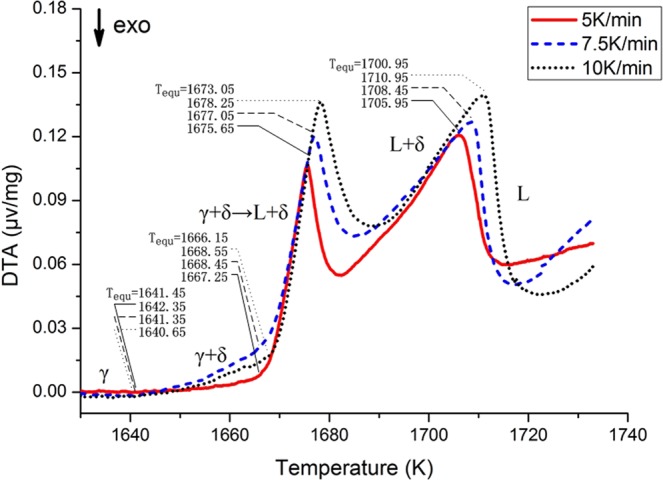
DTA experimental results for TWIP A.

Differential thermal analysis experiments were carried out on TWIP A samples at three different heating rates of 5 K/min, 7.5 K/min and 10 K/min. The result was shown in Fig. 1.It can be seen from the Fig. 1 that the DTA curves of the three experiments have a similar pattern, and crucial phase transformation points from DTA experiments with different heating rates have a close relationship to each other. As the heating rate increases, the DTA signal peak moves backward and the peak temperature increases. The liquidus temperature of the sample also increases with the increment of heating rate. Further analysis revealed that the phase transition temperature above the solidus temperature shows a completely linear relationship at different heating rates. If the heating rate is reduced to zero, it will become the equilibrium phase transition temperature; this can eliminate the error caused by the heating rate. Therefore, in order to make the research results more accurate, all the phase transition analysis results will take the equilibrium temperature in this study.

As we can see from Fig. 1, there are many signal peaks in the DTA curve of the TWIP A sample; it means that enthalpy changes associated with complex phase transitions occur in this process. There are four critical transition temperatures in the DTA curve. At the beginning, the baseline is very flat. The first deviation from the baseline is found at 1641.45 K (it is an equilibrium temperature, T_equ_), which indicates austenite to δ-ferrite transformation starts at 1641.45 K. Then a sharp DTA peak appears at 1666.15 K, and reaching the peak value at 1673.05 K. The onset temperature of the peak is the peritectic phase transition temperature (T_Perit_), and peak temperature (T_Perit end_) can be associated with the end of transformation δ + γ → L + δ. It can be found that the solidus temperature is accompanied by the enthalpy change, the peak of the DTA curve illustrates this phenomenon. Subsequently, the residual δ-ferrites continuously changes into a liquid phase, and finally, all of them become liquid phase. Therefore, the solidification pattern for the whole process is L → L + δ → L + δ + γ → γ + δ → γ.

**Figure 2:**
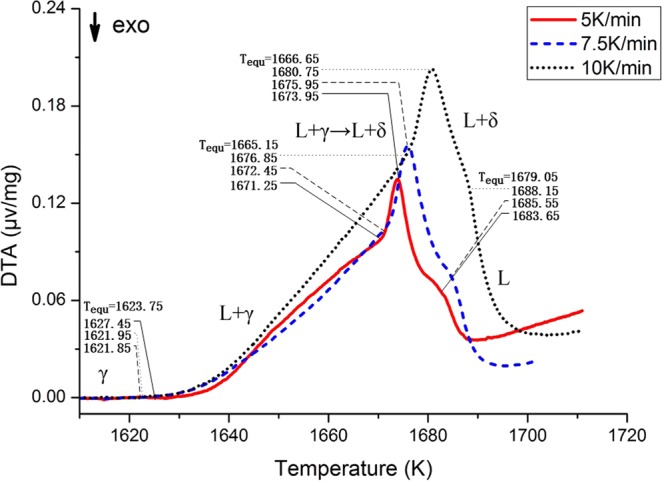
DTA experimental results for TWIP B.

Figure 2 shows there are four crucial transition points in DTA curve of TWIP B sample, and in the solid phase no austenite to δ-ferrite transformation took place. The deviation from the baseline at 1623.75 K means that the liquid phase begins to appear. When heating up to 1665.15 K, a sharp peak appears in the curve, and the austenite to δ-ferrite phase transformation occurs. At 1666.65 K, peak temperature phase transition is completed, only leaving δ-ferrite phase coexisting with the liquid phase. As the temperature increases, the δ phase gradually decreases. After 1679.05 K, all phases become liquid phase. Therefore, the solidification mode of this process is L → L + δ → L + δ + γ → L + γ → γ.

**Figure 3:**
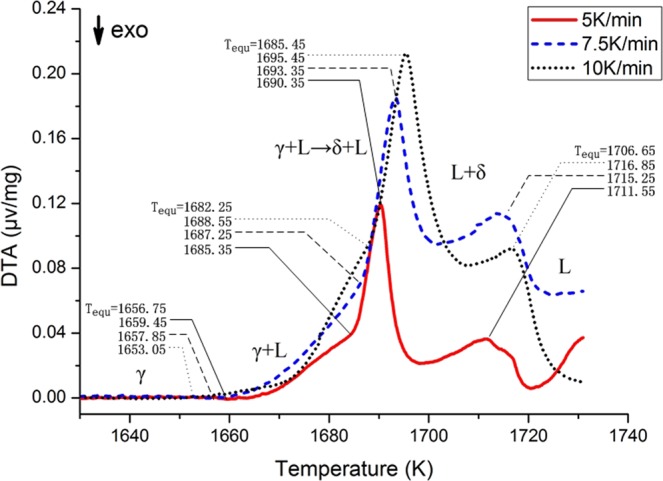
DTA experimental results for TWIP C.

As in Fig. 3, The DTA curve of TWIP C presented in Fig. 3 has a similar shape to that of TWIP B, yet details are different. When the temperature is heated to 1656.75 K, the DTA curve deviates greatly from the baseline and starts the γ → L + γ phase transition. A sharp peak begins at 1682.25 K, and it indicates peritectic reaction L + γ → L + δ occurs at this onset temperature. When it reaches the peak temperature 1685.45 K, the peritectic transition is completed and only δ ferrite and liquid left. If the temperature continues to rise above 1685.45 K, the δ ferrite in the steel decreases and the liquid phase increases, until all of them become liquid phases. This is a solidification pattern of L → L + δ → L + δ + γ → γ + L → γ.

### Crack sensitivity

**Figure 4:**
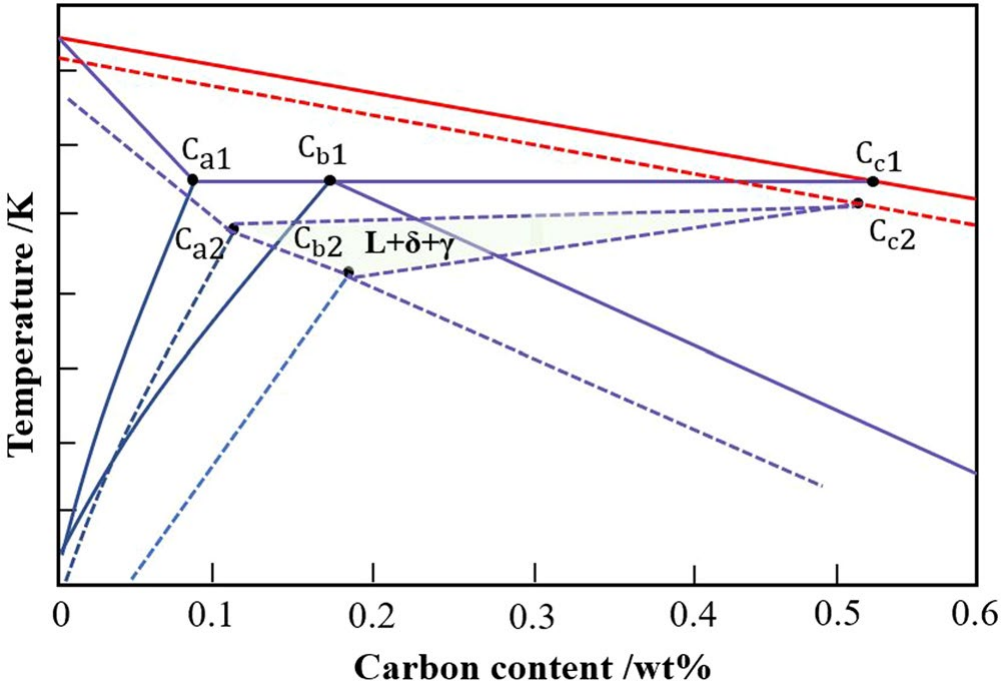
Pseudobinary Fe-C equilibrium phase diagram.

In the Fe-C equilibrium phase diagram, characteristic point C_a1_, C_b1_ and C_c1_ can divide the Fe-C phase diagram into four distinct carbon ranges. Range I is the left region of C_a1_, range II is between C_a1_ and C_b1_, range III is between C_b1_ and C_C1_, and range IV is the right side of C_C1_. In the pure Fe-C phase diagram, characteristic points C_a1_ and C_b1_ are in the positions of 0.09% and 0.17% carbon content respectively. However, general steel is multi-alloyed. These alloy elements can significantly affect the phase diagram and the positions of C_a1_ and C_b1_ points; it easily leads to the formation of an L + δ + γ ternary phase coexistence region^18^. This is different from the pure Fe-C phase diagram. The positions of characteristic points will become extremely hard to predict. In Fig. 4, it is clear to see that three characteristic points C_a1_, C_b1_, and C_c1_ in the pure Fe-C phase diagram have been shifted to the positions of C_a2_, C_b2_, and C_c2_. Moreover, a ternary phase coexistence region can be seen.

When the chemical composition is between C_a1_ and C_c1_ (or between C_a2_ and C_c2_), including range II and range III, the peritectic reaction will occur during solidification, and the steel is called peritectic steel. If the chemical composition of the steel is in the range II (between C_a1_ and C_b1_, or between C_a2_ and C_b2_), it is defined as hypo-peritectic steel.

**Figure 5:**
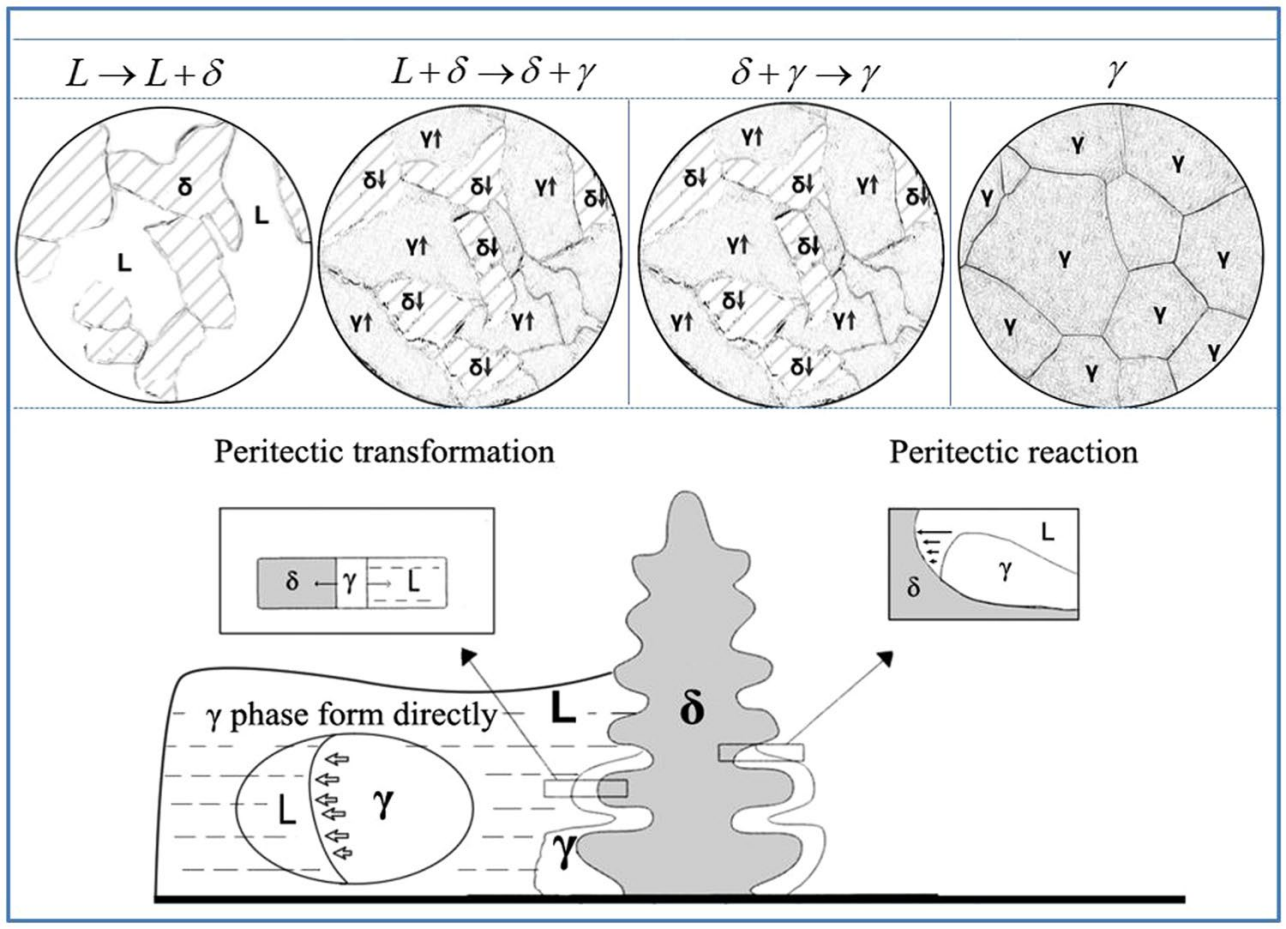
Mechanism of peritectic solidification^26,32^.

The composition of hypo-peritectic steel is within range II, and the peritectic reaction occurs during solidification. The solidification sequence is $L\to L+\delta \to \delta +\gamma \to \gamma$. The mechanism of the peritectic solidification process is shown in Fig. 5. As the temperature decreases and the solidification process continues, the δ phase exists in the liquid phase, and then the γ phase begins to appear. The primary γ phase grows along the δ / L interface. When the γ phase can separate the δ phase and the liquid phase to a certain extent, the γ phase not only grows rapidly with the dissolution of δ phase by the longrange solid phase diffusion, but also grows along the solid/liquid interface γ/L, or even can nucleate and grow directly in the liquid phase. Finally, the whole process of the peritectic transformation is completed.

The peritectic phase transformation of $\delta -Fe$ change into $\gamma -Fe$ is accompanied with final solidification process and end in the solid phase. At this stage, the high-temperature properties of the steel are very fragile. The shrinkage and thermal stress caused by the phase transformation can easily lead to defects such as hot cracks. Therefore, it is necessary to determine whether a new steel grade is within critical rangII. After solidification modes of the three different compositions TWIP steel were obtained from the DTA experiment, the solidification behavior of the TWIP steel can be analyzed to determine the crack sensitivity.

### TWIP steel solidification mode and crack sensitivity

Table 2 lists the critical phase transition temperatures and solidification mode of TWIP steel with three chemical compositions of TWIP A, TWIP B and TWIP C.

**Table 2:** Phase transition temperatures and solidification mode, K.

Sample	TWIP A	TWIP B	TWIP C
Phase transition temperatures	Between C_a2_ and C_b2_ Range II	Between C_b2_ and C_c2_ Range III	Between C_b2_ and C_c2_ Range III
T_Liquid_ (L → L + γ)	—	—	—
T_Liquid_ (L → L + δ)	1700.95	1679.05	1706.65
T_Perit-end_ (L + δ → L + δ + γ)	1673.05	1666.65	1685.45
T_Perit-start_ (L + δ + γ → L + γ)	—	1665.15	1682.25
T_Perit-start_ (L + δ + γ → γ + δ)	1666.15	—	—
T_Solid_ (L + γ → γ)	—	1623.75	1656.75
T_γ→δ_ (γ + δ → γ)	1641.45	—	—

There is a corresponding relationship between the solidification sequence of steel grade and the range area of the phase diagram. As can be seen from Table 2, it is known that TWIP A is in range II (between C_a2_ and C_b2_). The temperature range of peritectic reaction L + δ → γ is between 1673.05K and 1666.15K; δ → γ phase transition occurs during the time that the liquid phase has just disappeared. At 1641.45 K temperature, all the δ phase changes into γ phase. It can be identified as hypo-peritectic steel. When this type of TWIP steel is produced, the probability of occurrence of defects and cracks increases significantly. Great attention should be paid to the adjustment of production techniques to avoid serious production losses.

TWIP B belongs to range III (between C_b2_ and C_c2_) during solidification; there is always a liquid phase in the phase transition from δ phase to γ phase at 1666.65 K. The liquid phase can promptly fill voids caused by solidification shrinkage and is not prone to cracks. TWIP B and TWIP A have the analogous content of Mn, Si and Al elements, but different carbon content. It indicates that the carbon content has great influence on the solidification behavior and makes the solidification process change from range II to range III. At 1685.45 K, TWIP C has δ → γ phase transformation, and this process also has a liquid phase. It is also in range III during the solidification process. TWIP C has different carbon and manganese contents with TWIP A and TWIP B, but also has the same solidification sequence with TWIP B (range III). It suggests that both carbon and manganese have an effect on the solidification mode, which makes it difficult to determine the solidification behavior. If the composition is not suitable, it may be in range II with strong crack sensitivity. Therefore, it is essential to analyze solidification mode during composition design of new TWIP steel grades. The difficulty of production should also be taken into account in the design of components. Otherwise, the crack sensitivity of steel may be very high.

### Phase Diagram Thermodynamic Prediction Comparison

Thermodynamics software ThermoCalc is employed to predict phase transformation modes of TWIP A, TWIP B and TWIP C. In Table 3, critical phase transition temperatures predicted by ThermoCalc are evaluated by direct comparison with that measured from DTA results.

**Table 3:** Comparison of predicted and measured phase transition temperatures, K.

Sample	Compari-son	Solidificat-ion mode	T_γ→δ_	T_Solid_	T_Perit start_	T_Perit start_	T_Perit end_	T_Liquid_
			(γ → γ + δ)	(γ → L + γ)	(δ + γ → L + δ + γ)	(L + γ → L + δ + γ)	(L + δ + γ → L + δ)	(L + δ → L)
TWIP A	Predicted	Range II	1625.4	—	1643.85	—	1650.3	1693.0
	Measured	Range II	1641.45	—	1666.15	—	1673.05	1700.95
	Difference	Same	16.10	—	22.30	—	22.80	8.00
TWIP B	Predicted	Range III	—	1597.55	—	1636.25	1641.35	1668.75
	Measured	Range III	—	1623.75	—	1665.15	1666.65	1679.05
	Difference	Same	—	26.20	—	28.90	25.30	10.30
TWIP C	Predicted	Range III	—	1635.2	—	1659.1	1665.4	1701.0
	Measured	Range III	—	1656.75	—	1682.25	1685.45	1706.65
	Difference	Same	—	21.60	—	23.20	20.10	5.70

The thermodynamic software ThermoCalc predicts that the solidification mode of TWIP A is $L\to L+\delta \to L+\delta +\gamma \to \delta +\gamma \to \gamma$; the solidification behavior is in range II. It is precisely the solidification behavior of hypo-peritectic with high crack sensitivity, which is completely consistent with the results of DTA experiment.

For the specific characteristic points, the maximum difference between the predicted values and measured data of the four crucial characteristic points is 22.8 K, and the minimum difference is only 8 K. The prediction of TWIP B and TWIP C shows that both of them have the same solidification modes (range III).

The maximum difference between the predicted value and the measured result of TWIP C samples is 23.20 K. The result of predicting liquidus temperature is the most accurate. The minimum difference between predicted and measured values of TWIP B and TWIP C samples is 10.3 K and 5.7 K respectively. In general, the solidification behavior of Fe-Mn-C-Al TWIP steel can be predicted well by using thermodynamic software ThermoCalc. Compared with the experimental data, the predicted value by ThermoCalc is acceptable. The thermodynamic software ThermoCalc is proving to be particularly instructive for the development of Fe-Mn-C-Al TWIP steel.

## Conclusion

(1) TWIP steels with different chemical compositions were analyzed by differential thermal experiments. The solidification behavior of Fe-Mn-C-Al TWIP steel may be within the range II, and belong to hypo-peritectic steels. The δ → γ phase transition occurs during the time that the liquid phase has just changed into a fragile initial solid phase. This solidification mode can easily lead to cracks or other defects. In the production of this type of TWIP steel, the probability of occurrence of defects and cracks is greatly increased, and attention should be paid to the adjustment of production techniques to avoid production losses.

(2) By comparing TWIP steels with three different chemical compositions, it is found that TWIP A is a hypo-peritectic steel with high crack sensitivity, and in range II. But the solidification behavior of TWIP B and TWIP C is range III, and the crack sensitivity is low. TWIP B and TWIP A have a similar concentration of Mn, Si and Al elements, but different carbon content. It indicates that the carbon content has a great effect on the solidification behavior and makes the solidification process change from range II to range III. TWIP C has different carbon and manganese contents with TWIP A and TWIP B, but also has the same solidification sequence with TWIP B (range III). It suggests that both C and Mn have an effect on the solidification mode, which makes it difficult to determine the solidification behavior. If the composition is not suitable, it may be in range II with strong crack sensitivity. Therefore, it is essential to analyze solidification mode during the composition design of new TWIP steel grades, otherwise the crack sensitivity of steel may be very high.

(3) The comparison between the thermodynamic software ThermoCalc and the DTA experiment shows that the prediction results of the solidification mode of the Fe-Mn-C-Al TWIP steel by ThermoCalc are in agreement with that of DTA experimental results. With respect to the prediction of transformation temperature, the difference between the predicted temperature and the experimental results is small, and the maximum error is less than 2%. The thermodynamic software ThermoCalc is proving to be particularly instructive for the development of Fe-Mn-C-Al TWIP steel.

## Data Availability

All data generated or analysed during this study are included in this published article (and its Supplementary Information files).
